# A Rare Case of Dynamic Popliteal Artery Occlusion After Gunshot Injury with Reconstitution of Flow in the Frog-leg Position

**DOI:** 10.7759/cureus.2541

**Published:** 2018-04-26

**Authors:** Franz Toro-Pape, Boris Kumaev, Matthew Jenson, Jerry Matteo

**Affiliations:** 1 Department of Radiology, University of Florida College of Medicine, Jacksonville, USA; 2 Department of Interventional Radiology, University of Florida College of Medicine, Jacksonville, USA

**Keywords:** popliteal artery entrapment, dynamic maneuvers, digital substraction angiography, ct angiography, gunshot injury, vascular injury, penetrating injury, popliteal artery injury, frog-leg position

## Abstract

A 16-year-old male was transferred to our institution shortly after a gunshot injury to the right lower extremity. Physical examination was remarkable for two bullet entry points in the right posterior leg. A right lower extremity computed tomography angiogram (CTA) demonstrated a retained bullet fragment in the right popliteal fossa and a 10 centimeter (cm) in length occlusion of the proximal peroneal artery with reconstitution of flow distally. A diagnostic angiogram of the right lower extremity with the patient’s leg extended demonstrated lack of popliteal arterial flow immediately distal to the retained bullet fragment. Reconstitution of vascular flow was appreciated once the patient’s leg was placed in the “frog-leg” position.

## Introduction

Penetrating gunshot injuries with or without concomitant vascular injuries are not uncommon at level 1 trauma centers, including our institution. The role of our interventional radiology department in their management and treatment has become increasingly important throughout the last two decades. The development and seemingly exponential improvement of minimally invasive transcatheter techniques over the last two decades have enabled the treatment of arterial injuries and potentially life-threatening bleeds with the use of minimally invasive angiography and intervention. At our institution, upper and lower extremity vascular injuries are frequently encountered clinical scenarios by our interventional radiologists, vascular surgeons, and general surgeons. Most of these are secondary to blunt or penetrating trauma resulting in arterial injury and frequently treated with balloon occlusion, transarterial embolization, coiling, and endovascular repair with or without stenting.

High-energy gunshot wounds carry the highest risk of vascular injury, followed by penetrating trauma with concomitant extremity fractures [1]. The inguinal region, medial compartment of the thigh, and popliteal fossa are considered high-risk locations for penetrating and blunt injuries in the lower extremities due to the superficial location of vessels in these locations. If not recognized and treated rapidly, traumatic peripheral vascular injuries may rapidly lead to loss of limb and life [2-3].

Although traumatic arterial injuries, as well as the effects of aging and mechanical stressors on the popliteal artery, have been well-documented in the literature [4], dynamic compression of the popliteal artery by a retained bullet fragment or other foreign bodies on diagnostic angiography is a rarely encountered clinical phenomenon and, to our knowledge, not yet reported in the literature. A focused literature search of the PubMed electronic database using the popliteal artery as a major topic in the Medical Subject Heading (MeSH) with additional MeSH subheading terminology, such as "digital subtraction angiography" (DSA), "angiography", "provocative DSA", "provocative maneuvers", "bullet", "ballistic", "angiography", "limb flexion", and "limb extension" was conducted, which did not yield a case similar to ours. 

## Case presentation

The patient is a 16-year-old male without a significant past medical history who was transferred to our institution after a gunshot injury to the right lower extremity. On physical examination, two bullet entry points were evident at the right popliteal fossa and dorsal soft tissues of the distal right leg. Initial radiographs were negative for fractures or dislocation.

Computed tomography angiography (CTA) demonstrated a retained bullet fragment within the popliteal fossa abutting the dorsal aspect of the popliteal artery. An 8 millimeter (mm) soft tissue density abutting the medial aspect of the popliteal artery was also identified, concerning for either a small pseudoaneurysm or short segment intramural hematoma. Streak artifact from the retained bullet precluded adequate assessment of this region. The peroneal artery demonstrated a 10 centimeter (cm) occlusion 2.5 cm distal to its origin but reconstituted distally at the level of the mid-tibia. The anterior and posterior tibial arteries were both normal in appearance and patent. The dorsalis pedis artery was unremarkable. There was subcutaneous emphysema throughout the deep and superficial posterior compartment of the knee and throughout the medial aspect of the leg.

**Figure 1:**
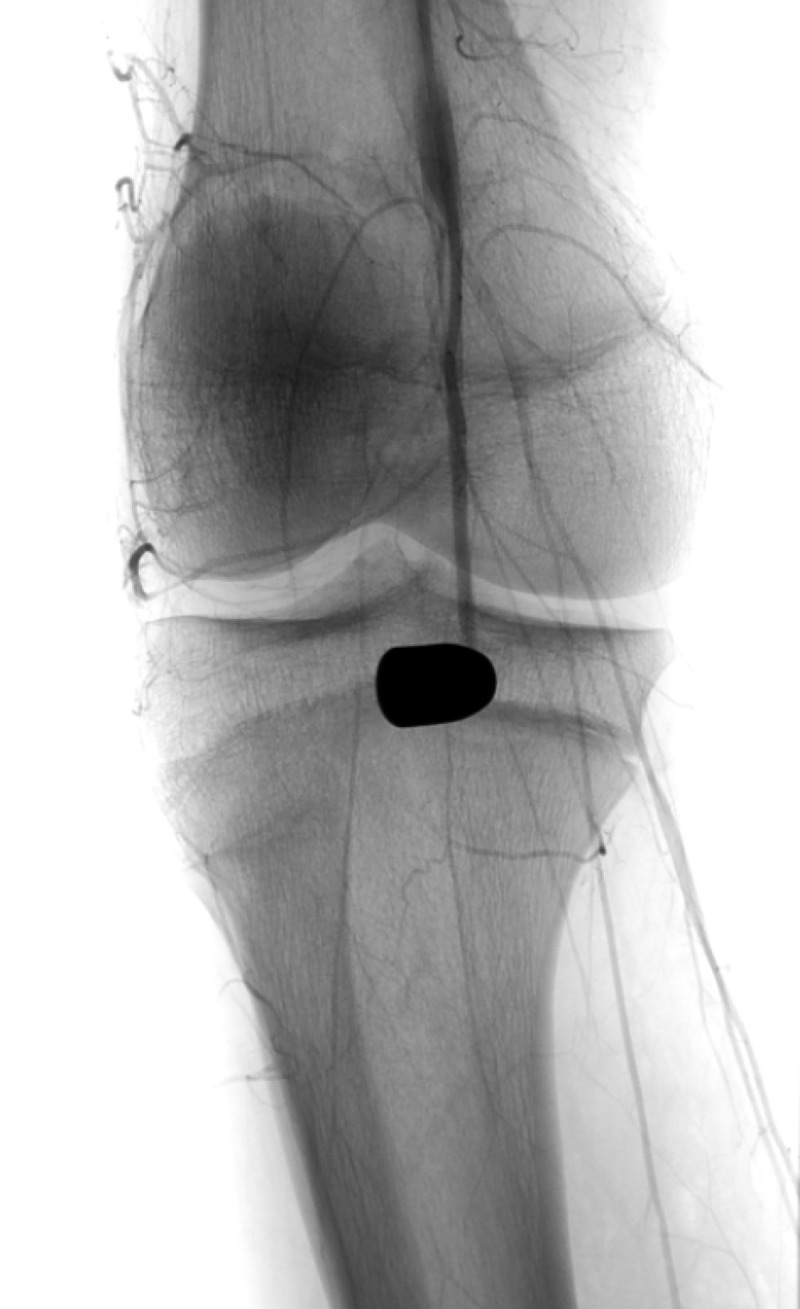
Right lower extremity angiography at the level of the knee with the patient’s leg held in extension There is a retained bullet fragment in the popliteal fossa and abrupt cessation of vascular enhancement in the above the knee popliteal artery.

**Figure 2:**
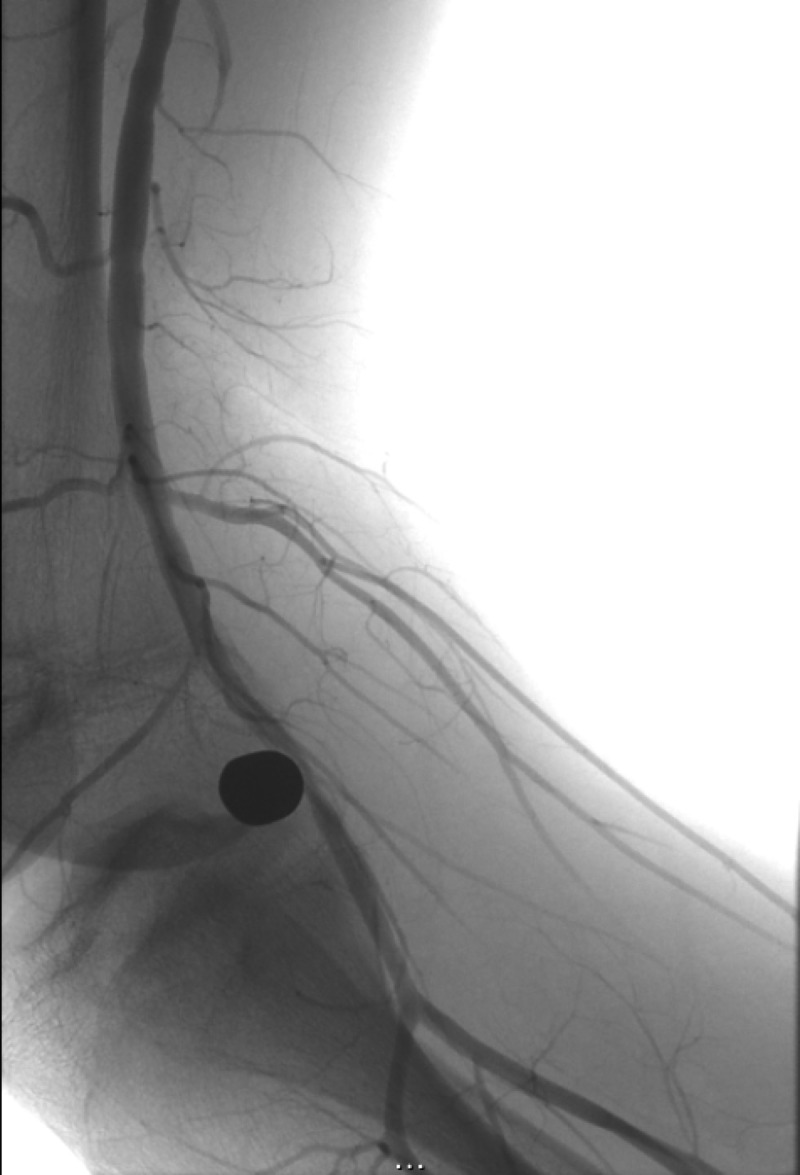
Right lower extremity angiography at the level of the knee with the patient's leg in the frog-leg position There is reconstitution of flow within the popliteal artery down to the level of the tibial-peroneal trunk when the patient's leg was placed in the frog-leg position.

The patient was taken to the interventional radiology suite and a right lower extremity diagnostic runoff angiogram was performed. Initial images obtained with the patient’s leg held in extension demonstrated abrupt cutoff of the popliteal artery immediately adjacent to the bullet fragment (Figure 1). We then proceeded to reposition the patient’s right leg in the “frog-leg position”. A second diagnostic runoff angiogram was then performed demonstrating mild short segment narrowing of the popliteal artery immediately adjacent to the bullet fragment but with reconstitution of flow down to the level of the tibial-peroneal trunk (Figure 2). The anterior and posterior tibial arteries demonstrated patency on both the extension and frog-leg positions. It was concluded that leg straightening/extension was contributing to extrinsic compression and subsequent dynamic occlusion of the popliteal artery secondary to the bullets close proximity to the vessel.

The patient was subsequently taken to the operating room for exploration and removal of the bullet fragment. An intraoperative angiogram was negative for popliteal artery vascular injury, and the anterior and posterior tibial arteries were both patent without evidence of injury or flow-limiting stenosis.

## Discussion

The superficial femoral artery is a continuation of the common femoral artery after it gives off the deep femoral branches in the upper thigh. It continues its caudal course primarily throughout the medial aspect of the thigh, eventually becoming the popliteal artery past the adductor hiatus. In the lower extremities, arterial injuries below the adductor hiatus result in amputation more often than do injuries in any other site [5]. The management and the importance of quick diagnosis of lower extremity arterial injuries have been well-documented in the literature. Recognition of a vascular injury and expeditious transport to repair are imperative to avoid loss of limb and life.

Provocative maneuvers in order to evaluate the popliteal artery have been primarily described in cases of popliteal artery entrapment syndrome in patients undergoing CTA; however, to our knowledge, none have been described in the setting of trauma and DSA. A recent study highlighted the management and approach in the diagnosis of popliteal artery entrapment syndrome in a small sample of 15 patients. The workup included a two-part CTA first performed with the patient's knee in a neutral position, and a second with the knee hyperextended with active plantar flexion in order to contract the medial head of the gastrocnemius muscle. A total of 10 patients with mild stenosis and an additional two patients with severe stenosis during active plantar flexion were identified who had an otherwise negative exam when the knee was scanned in the neutral position [6]. This study highlights the importance of provocative maneuvers in the radiologic diagnosis of vascular entrapment syndromes. 

A systematic review and meta-analysis evaluating the diagnostic accuracy of CTA in detecting arterial lesions in patients with suspected arterial injury of the upper or lower extremity showed that CTA is an accurate modality for evaluating arterial lesions in patients with extremity trauma and can replace digital subtraction angiography [7]. Although in full agreement that CTA is an invaluable diagnostic modality and essential for expeditious recognition of vascular injuries, DSA has the added benefit of intervention and extremity manipulation, as highlighted in our case. DSA is also less technically dependent, particularly in cases where the bolus may be deemed suboptimal for the evaluation of disease. This is of particular importance in the setting of trauma where time to diagnosis and subsequent intervention is of the essence.

## Conclusions

Our case highlights the importance of dynamic maneuvers during diagnostic angiography in order to assess patency of potentially injured or occluded vessels when the extremity of interest is held in neutral position or fixed in the position of initial presentation. We recognize that provocative maneuvers are sometimes clinically not feasible due to concomitant secondary injuries and other related patient limitations frequently encountered in cases of penetrating or blunt extremity trauma.
